# Enhancing encapsulation of filarial antigen *Brugia malayi* thioredoxin in nano-liposomes: The role of lecithin composition

**DOI:** 10.5599/admet.2089

**Published:** 2023-11-30

**Authors:** Malathi Balasubramaniyan, Vimalraj Vinayagam, Moni Philip Jacob Kizhakedathil, Kaliraj Perumal

**Affiliations:** 1 Department Centre for Nanoscience and Technology, Anna University, Guindy, Chennai 600025, Tamilnadu, India; 2 Department of Biotechnology, Vignan's Foundation for Science, Technology & Research University, Vadlamudi 522213, Andhra Pradesh, India; 3 Department of Chemistry, Bannari Amman Institute of Technology, Sathyamangalam, 638401, Tamilnadu, India; 4 Department of Biotechnology, Bannari Amman Institute of Technology, Sathyamangalam, 638401, Tamilnadu, India; 5 Centre for Biotechnology, Anna University, Guindy, Chennai 600025, Tamilnadu, India

**Keywords:** liposome, lymphatic filariasis, thioredoxin, egg phosphatidylcholine, cholesterol

## Abstract

**Background and purpose:**

Lymphatic filariasis is a debilitating infectious disease prevalent in endemic areas, necessitating the development of an effective vaccine for eradication. Although recombinant vaccine candidates have been deemed safe, they often fail to provide sufficient protection, which can be overcome by encapsulating them in nano-liposomes. In this study, we have optimised the liposomal composition for enhanced stability and encapsulation of filarial antigen *Brugia malayi* thioredoxin (Bm-TRX).

**Experimental approach:**

Nano-liposomes were prepared using egg phosphatidylcholine (EPC) and cholesterol via thin-film hydration, followed by sonication and characterizing. Encapsulation efficiency was optimised using different weight ratios of EPC to cholesterol (8:2, 7:3, and 6:4) and total lipid (EPC+Cholesterol) concentration to antigen Bm-TRX (10:1, 10:2, and 10:3) followed by release kinetics study.

**Key results:**

Optimised parameters yielded spherical liposomes measuring 209 nm in diameter with narrow polydispersity. Our findings demonstrated the highest encapsulation efficiency of 70.685 % and stability of 10 hours for an EPC to cholesterol weight ratio of 7:3. The *in silico* study proved the antigenic nature of TRX.

**Conclusion:**

The liposomal formulations loaded with TRX, as optimized in this study, hold promise for improving antigen efficiency by enhancing stability, bioavailability, and prophylactic effects by acting as immune potentiators.

## Introduction

Lymphatic filariasis (LF) is a vector-borne morbid infectious disease caused by filarial worms *Wuchereria bancrofti* (Wb) and *Brugia malayi* (Bm). LF creates profound disfiguring and socio-economic problems along with health complications in developing countries [1]. These nematode infections are the world's second leading cause of permanent and long-term disability [2]. Tragically, 120 million people are affected by LF, and still, 1.39 billion are prone to infection risk [3]. The current prevention and prophylaxis involve diagnosis, inhibition of transmission, morbidity control and disability avoidance. Interruption of transmission for the population at risk is achieved by mass drug administration of albendazole plus either diethylcarbamazine or ivermectin [4,5]. But, the complex life cycle of the filarial worm makes the pharmacological efficacy limited. Also, there is an evolving issue of drug resistance and rapid re-emergence of LF in endemic areas where transmission cannot be interrupted. These issues led to new intervention strategies for identifying and successfully developing several recombinant proteins from filarial worms as effective vaccine candidates for prophylactics. Thioredoxin (TRX), exhibiting antioxidant properties found in nematodes, has proved to play a crucial role in parasite’s regulatory and immune-modulatory evasion mechanisms [6]. The recombinant TRX from *Brugia malayi* (Bm-TRX) has provided 63 % protection in murine animal models [7]. But, this recombinant antigen has failed to produce a high degree of protection, unlike irradiated infective third-stage larvae (L3) [8,9]. These issues might be mainly due to the degradation of antigens and, thereby, leading to low availability *in vivo*. Therefore, to increase antigen stability and enhance sustained release, they can be encapsulated in carriers such as liposomes [10,11].

Liposomes are carriers with sizes ranging from nano to micron scale and are formed by combinations of various phospholipids and cholesterol [12]. These lipids are obtained from natural sources, such as chicken egg yolk or soybeans, and therefore are considered safe (GRAS) because they are biodegradable, biocompatible and nontoxic [13,14]. They have proved to be efficient carriers as well as immune potentiators by inducing balanced T_H_1 & T_H_2 responses [15-17]. Simple liposomes formed from phosphatidylcholine and cholesterol, the natural composition of the human cell membrane, stimulate efficient immune response [15,18]. Since liposomes are a stable carrier, they can enhance the stability and bioavailability of filarial antigens, thereby improving the prophylactic behaviour of TRX.

The main objective of this study is to develop stable liposomal vehicles using EPC and cholesterol in different weight ratios. These nano-liposomes will be characterized and tested for their encapsulation efficiencies by incorporating antigen Bm-TRX in various lipid:antigen ratios. Additionally, the stability of the liposomes will be assessed through an analysis of the antigen release kinetics.

## Experimental

### Production of filarial antigens

The *Brugia malayi* pRSETB-TRX construct was overexpressed in *E. coli* (GJ1158) strain using NaCl induction and the recombinant protein was purified by ion-exchange chromatography using Q-sepharose (Amersham Pharmacia Biotech) under non-denaturing conditions [7].

### Preparation of nano-liposomes

The empty liposome was prepared using a modified thin film hydration technique. Briefly 1 mg mL^-1^ of egg phosphatidylcholine (EPC) from Sigma-Aldrich, USA, dissolved in chloroform, was taken in a clean, moisture-free vial and purged with nitrogen gas to remove the solvent. The thin film formed is then hydrated with phosphate buffer saline (PBS, pH 7.4). The vial was stirred continuously at 60 °C for 30 min. The solution was then probe sonicated for various cycles (5, 10, 15, 20, 25) to obtain nano-scaled liposomes. The sonication was carried out at 30 kHz processing frequency with a cycle duration of 30 s on and 30 s break to cool down the sample. The heat released by sonication was cooled by immersing the vial in a beaker full of ice water.

### Size and morphology characterization of liposomes

The size and uniform distribution of prepared liposomes were confirmed using dynamic light scattering (DLS) instruments. The transmission electron micrographs of lyophilized nano-liposomes formed with an optimised cycle of sonication were re-suspended in double distilled water and 1 μL of this dispersion was placed on the grid, air dried and then loaded into the specimen chamber. The TEM images were obtained using JEOL JEM -200CX at 60kV.

### Filarial antigen loading

In order to determine high encapsulation efficiency, various concentrations of EPC and cholesterol (Ch) in weight ratios of 10:0, 9:1, 8:2, 7:3, 6:4 of 5:5 were analysed. The phospholipids (EPC:Ch) of various combinations were taken in sterile vials and purged. The various weight ratios of phospholipids were evaluated for their efficacy in encapsulating recombinant protein Bm-TRX in the following weight ratios: 10:1, 10:2 and 10:3. Antigens in PBS (pH 7.4) were taken and added to the purged lipids. It was then stirred for 30 min at 60 °C and sonicated to form TRX-loaded nano-liposome (TL).

### Determination of encapsulation efficiency

The sonicated liposomes were centrifuged at 10,000 rpm at 4 °C for 10 min to separate unbound TRX from the loaded liposomes. The liposomes were washed with PBS (pH 7.4) until no protein was found in the supernatant. The presence of TRX in supernatant was estimated using the BCA method. All the experiments were carried out in triplicates. The encapsulation efficiency was estimated using the formulae given in Equation (1).



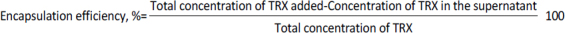

(1)


### Release kinetics

1 mg of lyophilised TL having good encapsulation was suspended in 1 mL PBS (pH 7.4) and incubated at 37 °C under mild agitation in different containers. At a predetermined time interval, a container was taken out and centrifuged at 10,000 rpm for 10 min to separate the released antigen from encapsulated liposomes. The release was determined using the formulae given in Equation (2).



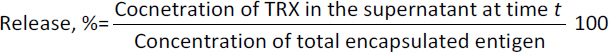

(2)


### In silico analysis

#### Protein retrieval, modelling and validation

For the current study, the protein thioredoxin from *Brugia malayi* was retrieved from NCBI - protein database, whose accession ID is AAM51563.1. The FASTA sequence was used for further analysis. In order to generate the three-dimensional model of the protein, ROBETTA online server was used. *Ab-initio* modelling of the selected protein was performed [19]. Further, the three-dimensional model was validated using Ramachandran plot analysed using SAVES V6.0 and Molprobity server. The comparative identity of thioredoxin sequence from *Brugia malayi* was done against non-redundant protein sequences against Homo sapiens present in the other databases with the parameters of (Expected Threshold:0.05, sequence identity: ≤45 %) using BLASTP server [20].

#### Epitope prediction and its properties

In order to identify B cell-specific epitopes, an online tool, ABCPred server, was used. The prediction was made with a Window length 14 and an epitope specificity threshold 0.51. The antigenic properties of the predicted epitopes were evaluated using the VaxiGen 2.0 server with Target Organism as a parasite and a cut-off score of 0.6 [21]. The predicted epitopes were also checked for allergenicity to prevent undesirable immune reactions. The prediction was carried out using the Allergen FP v.1.0 server [22,23]. Conformational epitope prediction of the protein modelled was carried out using an epitope 3D server [24].

#### Prediction of MHC class I and MHC class II epitopes

To predict the peptides binding to Major Histocompatibility Complex1 (MHC 1), the online server Net MHC 4.0 was used [25]. The predicted epitope sequences were given as input with a peptide length of 14 mer and a representative set of reference alleles belonging to MHC 1 consisting of Human Leukocyte Antigens (HLA- A, B, and C) based on a trained dataset. On the other hand, the peptide binding to Major Histocompatibility Complex 2 (MHC-2) was done using the Net MHC II 2.3 server. The epitope sequences were set as input with parameters (peptide length-14 and representative alleles- DP, DQ, DR). The binding affinities were sorted by setting a threshold for strong binders at 2 and weak binders at 10. The rank of binding affinity values <1500 nM was selected for further analysis [26].

### Immune simulation of the protein

The C-IMM SIM server was used to perform the immune simulation of the thioredoxin for assessing the immunogenicity and immune response attributes. The immunogenic profile was simulated in three different anatomical regions (bone marrow, thymus, and lymph node) in mammals. The entire simulation ran for 1050 time steps with the administration of three peptide injections at an interval of two weeks using a time step set at 1, 42, 84 (individual time step is 8 hours and time step 1 is injection at time = 0). The three injections comprising 1000 vaccine proteins were used. The administration of the vaccine injection was used to calculate the Antibody and Cytokine production immune responses of Helper Cells, B-cells, Natural killer cells and antigen-presenting cells [27].

### Statistics

All the results were done in triplicates and are expressed as mean ± SD. Analysis of variance (One-way Anova) of data was performed for statistical significance (*p* < 0.05), followed by Tukey’s test for individual column comparison. In all circumstances, statistical analysis was conducted using the GraphPad Prism software Version 5.

## Results and discussion

### Expression and purification of filarial antigen

The recombinant filarial antigen TRX without histidine tag is expressed as 20 kDa protein and purified using ion-exchange chromatography.

### Synthesis and characterization of nano-liposome

**Figure 1.**
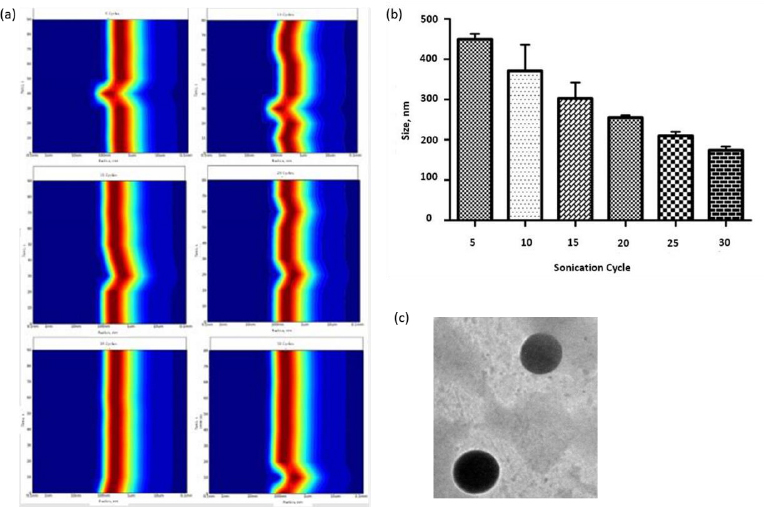
(a) Effect of sonication on uniform distribution of liposomes. The DLS uniformity study shows that the 25^th^ cycle produced evenly distributed liposomes. (b) The increase in the sonication cycle reduced the size of the liposomes. (c) TEM images of liposomes were produced with EPC at 25 cycles of sonication.

Liposomes were prepared by thin film hydration technique. The thin film, when hydrated with an aqueous buffer, spontaneously swells and folds to form liposomes. As this technique forms heterogeneous-sized liposomes with micron range diameter, the size is further reduced by various sonication (5, 10, 15, 20, 25, and 30) cycles. The size was determined using DLS. Though sonication led to the formation of nano-scaled liposomes for the various cycles (449.79 ± 13.47, 371 ± 64.89, 302.87 ± 39.56, 255.09 ± 5.65, 209.86 ± 9.79 and 174.16 ± 9.02 nm), uniform distribution was obtained for 25 cycles of sonication as shown in the Figures 1a and b. As evident from Figure 1(a), sonication plays a crucial role in the size and distribution of the vesicles. Earlier studies have suggested that the higher energy input results in control of final vesicles and homogeneity via mechanical collision [28-30]. This collision causes breaking and making processes, leading to smaller size. And these findings correlate with our present study. Thus, we infer that the optimised sonication cycle of 25 led to the formation of narrow polydispersity nano-liposomes, which were taken for further characterization using TEM. The TEM image (Figure 1c) further confirms the size of nano-liposomes. The image also revealed the spherical morphology and narrow size of the optimised nano-liposomes.

### Filarial antigen encapsulation

**Figure 2.**
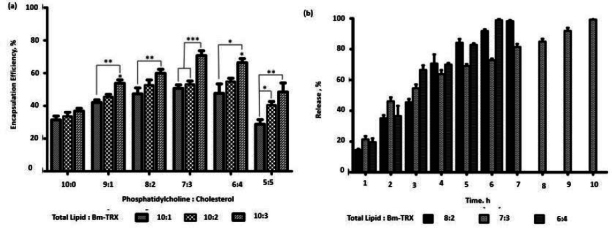
(a) Effect of cholesterol and phopholipid : TRX weight ratio on encapsulation efficiency. (b) TRX-release kinetics from nano-liposomes.

The EPC and cholesterol in ratios (10:0, 9:1, 8:2, 7:3, 6:4, 5:5) with a total lipid quantity of 10 mg was used to encapsulate TRX in various phospholipid to antigen ratio (10:1, 10:2, 10:3). The liposomal composition plays a significant role in its antigen loading and stability. As shown in Figure 2a, the encapsulation efficiency of TRX was influenced by the presence of cholesterol. Encapsulation efficiency increased with increase in cholesterol and antigen weight ratio. Increase in encapsulation was observed till 7:3 (phospholipid: cholesterol) weight ratio and 10:03 (phospholipid: TRX) weight ratio.

The release of antigen from different weight ratios of EPC: cholesterol (8:2, 7:3, 6:4) showed comparatively stable release in 7:3 compositions. The liposome consisting of cholesterol (7:3) showed significantly high encapsulation (*p*>0.05) when compared to EPC with no cholesterol. Further increase of cholesterol diminished the antigen loading efficiency of liposomes. The lipid to TRX 10:3 ratio had high encapsulation efficiency. Enhancement of cholesterol interferes with the tight packing of lipids and induces membrane fluidity. This results in better distribution of the aqueous phase and, thereby, amplifying protein encapsulation. Earlier study has also reported the role of cholesterol in incrementing encapsulation [31]. The role of electrostatic interaction between the bovine serum albumin and lipid surfaces in increasing the encapsulation of the former has also been reported [32]. We, therefore, infer that along with entrapment in the hydrophilic compartment, there is an electrostatic interaction between the negatively charged TRX at neutral pH with the positively charged amine in the bilayer composition of liposome, thereby resulting in significantly higher encapsulation efficiency in a 10:3 ratio. Thus, we validate the 10:03 ratio of phospholipid: TRX as an optimised proportion for further application of nano-liposomes as vaccine carriers.

### Release kinetics

The optimised lipid:TRX ratio (10:3) was encapsulated in a different combination of EPC:Cholesterol (8:2, 7:3, 6:4) and investigated the release kinetics. The percentage release of TRX was found to be 35.0 %, 46.16 and 36.59 % with EPC: Cholesterol ratios (8:2, 7:3, 6:4), respectively, in the initial 2 h (Figure 2b). With initial burst release, the ratio of 7:3 had stable and prolonged release compared to the other ratios. Nearly 99.2 % was released in 10 hours. TRX proteins are amphipathic by nature; they associate with the lipid bilayer, and at the same time, their hydrophilic tails may project on the surface [33]. The release of electrostatically surface adsorbed antigens gives the initial burst release when incorporated into PBS. But there is a stable release after that. This stability is postulated to the fact that the incorporation of cholesterol in EPC significantly reduces the release of proteins from liposomes [10]. It leads to a comparatively gradual release of TRX, along with the disintegration of lipid lamellae.

### Sequence retrieval, modelling and validation

**Figure 3.**
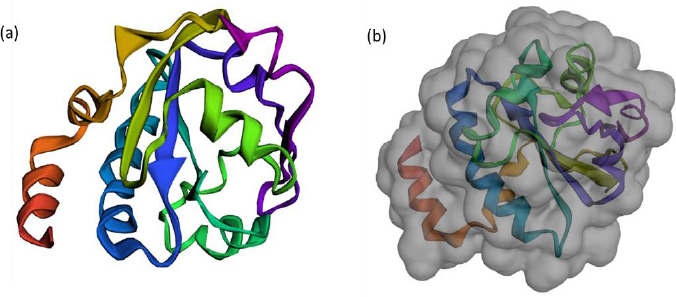
(a) 3-D model of the protein thioredoxin modelled using Robetta server (b): 3-D surface model of the protein thioredoxin modelled using Robetta server

The FASTA sequence of protein thioredoxin (accession ID is AAM51563.1) from *Brugia malayi* was retrieved from NCBI - protein. The sequence was subjected to pBLAST analysis against a non-redundant protein sequence database, Organism - Homo sapiens. Based on the threshold of sequence identity ≤45 %, it could be derived that the protein was highly conserved in *Brugia malayi* and doesn't share homology with Homo sapiens. The three-dimensional model of the protein was constructed using ROBETTA. *Ab-initio* modelling was performed using the default parameters (Figures 3a and 3b).

Five models were generated and the best model was selected based on the analysis of Ramachandran plots. Three-dimensional modelling of the proteins gives an idea regarding the key elements in a protein. It also helps in understanding the interaction and the molecular dynamics of the protein [34].

**Figure 4.**
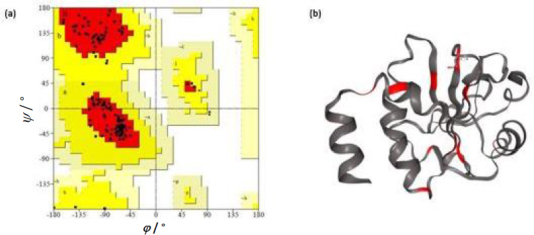
(a) Ramachandran plot of the modeled protein showing the amino acid residues are in the allowed region (b): Conformational epitopes predicted using epitope3D.

The Ramachandran plot analysis gives an idea to select the best model based on several parameters, such as the presence of outliers in the number of residues present in the favored and allowed regions. Based on the Ramachandran plot analysis, the best model had 98.6 % (141/143) of all residues in the 98 % favored regions and 100 % (143/143) of all residues in the allowed regions (>99.8 %) with no outliers (Figures 4a and 4b).

### B cell and T cell epitope prediction and its properties

B cells can identify specific epitopes on the proteins and trigger the differentiation into plasma cells, which produce antibodies that help neutralize and opsonization.

The B-cell epitopes were predicted using ABCpred online with a window length 14 and epitope specificity threshold of 0.51. Fifteen such B cell epitopes were predicted and tabulated in Table 1. The epitopes were ranked based on the predicted scores. The predicted epitopes were further analysed for their antigenicity and allergenicity. Vaxigen server was used to predict the antigenicity of the given epitopes.

**Table 1.** The antigenicity of predicted B cell epitopes All the epitopes were found to be non-allergen.

B cell epitopes				Antigenicity	
Rank	Sequence	Start position	Score		
1	NKYEVAGIPMLIVI	101	0.86	0.3197	Non - Antigen
2	YYVPFGSSEIEKLK	87	0.76	-0.1235	Non - Antigen
2	ALYFSAHWCPPCRQ	31	0.76	0.3652	Non - Antigen
3	LDHSEEDLNNYVKE	68	0.75	0.6018	**Antigen**
4	PMLIVIKSDGNVIT	109	0.74	1.1595	**Antigen**
5	DQFEIVFVSLDHSE	59	0.72	-0.0136	Non - Antigen
6	NLKKADGTVKKGSD	9	0.69	0.0201	Non - Antigen
6	ADLLANINLKKADG	2	0.69	0.161	Non - Antigen
7	KSDGNVITKNGRAD	115	0.61	1.2459	**Antigen**
8	CRQFTPILKEFYEE	42	0.59	0.9215	**Antigen**
9	VKESHGNWYYVPFG	79	0.57	0.2245	Non - Antigen
9	DLNNYVKESHGNWY	74	0.57	0.6565	**Antigen**
9	HWCPPCRQFTPILK	37	0.57	-0.0531	Non - Antigen
10	SSEIEKLKNKYEVA	93	0.56	0.1791	Non - Antigen
11	KGSDALANKKVVAL	19	0.54	**-0.0033**	Non - Antigen

The threshold was set at 0.51. Based on the prediction results, it was observed that five epitopes, *i.e.* LDHSEEDLNNYVKE, PMLIVIKSDGNVIT, KSDGNVITKNGRAD, CRQFTPILKEFYEE and DLNNYVKESHGNWY were antigenic in nature. Allergenicity was predicted using the Allergen FP server and the epitopes were recognised as an allergen/non-allergen based on the Tanimoto index. From the results, it was seen that all the predicted B cell epitopes were non-allergenic. Identification of antigenic regions is fundamental for designing vaccines and for immunotherapies. It is key to identify the conformational epitopes in a protein. Epitope 3D server was employed to analyse the conformational epitopes from a three-dimensional protein structure (Figure 4b).

The shortlisted epitopes were analysed for their affinity to bind with various MHC I and MHC II alleles using Net MHC servers. Binding of peptides to MHC I and MHC II receptors allows the identification of the peptides by Cytotoxic T cells and Helper T cells, respectively. MHC I class receptors are present on the surface of all nucleated cells, which helps identify endogenous antigens and presents these antigens to Cytotoxic T cells. Whereas MHC II is involved in the detection and binding of exogenous antigens and presents these antigens to Helper T cells. The strong and weak binders associated with class 1 and 2 MHC alleles are noted. There were six MHC Class I alleles found associated with the selected epitopes. The alleles are HLA-A0101, HLA-A2501, HLA-A2602, HLA-A8001, HLA-B1502 and HLA-B1503. Similarly, 24 MHC Class II alleles were found to be associated with selected epitopes. The alleles are DRB1_0301, DRB1_0401, DRB1_0403, DRB1_0404, DRB1_0405, DRB1_0701, DRB1_0802, DRB1_1101, DRB1_1302, DRB1_1501, DRB1_1602, DRB3_0101, DRB3_0301, DRB4_0101, DRB5_0101, HLA-DPA10103-DPB10301, HLA-DPA-10103-DPB10401, HLA-DPA10201-DPB10101, HLA-DPA10201-DPB10501, HLA-DPA10301-DPB10402, HLA-DQA10102-DQB10501, HLA-DQA10301-DQB10302, HLA-DQA10401-DQB10402 and H-2-IAk.

### Immune simulation of the protein

**Figure 5.**
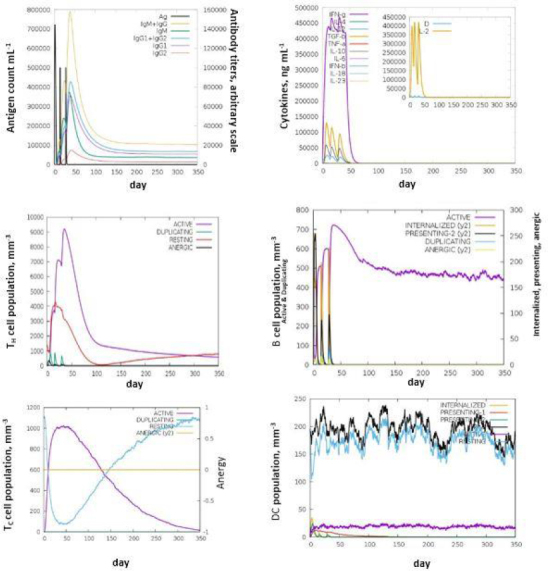
C-IMM SIM simulation of immune response. (a) Antigen and immunoglobulins. Antibodies are subdivided per isotype. (b) Cytokines. Concentration of cytokines and interleukins. D in the inset plot is a danger signal. (c) CD4 T-helper lymphocytes count sub-divided per entity-state (*i.e.* active, resting, anergic and duplicating), (d) B lymphocytes: total count, memory cells, and subdivided in isotypes IgM, IgG1 and IgG2, (e) CD8 T-cytotoxic lymphocytes count per entity-state. (f) Dendritic cells. DC can present antigenic peptides on both MHC class-I and class-II molecules. The curves show the total number broken down to active, resting, internalised and presenting the antigen.

The C- IMM SIM measures the effective immune responses of the vaccine construct. It predicts immune cell memory and its lifespan based on the cell's state. The server results showed consistent immune responses, with secondary and tertiary responses showing increased activity compared to the primary response. High levels of immunoglobulins (IgM, IgM + IgG, IgG1+IgG2, IgG1, and IgG2) indicate an immune response associated with humoral immunity. Research suggests that IgG1, IgG2, and IgA antibodies enhance bacterial clearance, while IgG3 and IgG4 inhibit opsonic activity and can cause tissue damage (Figures 5a-5f).

The decrease in antigen concentration suggests an increase in B-cell isotype IgG1 and B memory cells. The responses from memory cells indicate a strong response in cytotoxic and helper T cell populations. Additionally, high levels of IL-12, IFN-γ, and T_H_1 populations were observed during multiple exposures, suggesting the antigenic nature of the protein [35].

## Conclusions

Narrow polydispersed liposomes with a mean diameter of 209 nm were prepared using Thin film hydration followed by sonication (25 cycles). Filarial antigen, TRX, was successfully encapsulated into the liposomes. The best encapsulation was observed at a lipid to TRX ratio of 10:3. On comparing various ratios of egg phosphatidylcholine to cholesterol, the ratio of 07:3 showed high encapsulation efficiency and release up to 10h at physiological pH. The presence of an optimised cholesterol level led to higher liposome stability, thereby lowering antigen release. The i*n silico* work indicated that predicted epitopes in TRX had high antigenic properties. Overall, we conclude that these optimised formulae may be used as a vaccine carrier for lymphatic filariasis.
